# 2019 Coronavirus Disease, Beware of Psychogenic Issue

**DOI:** 10.1017/dmp.2020.153

**Published:** 2020-05-18

**Authors:** Qianshi Zhang, Xin Qi

**Affiliations:** Department of Spine Surgery, The Second Xiangya Hospital, Central South University, Changsha, Hunan, China 410011; Department of Ophthalmology, The Second Xiangya Hospital, Central South University, Changsha, Hunan, China. 410011

**Keywords:** conjunctivitis, COVID-19, psychogenic issue, public health

An ongoing outbreak of pneumonia associated with a novel coronavirus disease (COVID-19)^1,2^ was first reported in Wuhan city, Hubei Province, China,^3^ and has now spread throughout the country. Affected patients were geographically linked with a local wet market as a potential source,^3^ and it has been indicated that it could be transmitted by respiratory or person-to-person contact. Symptoms include fever, upper or lower respiratory tract symptoms, or diarrhea,^3,4^ and a few patients have complained of conjunctivitis at the early stage of the 14-day incubation period.^5^ Health care workers all over the country are fiercely working to prevent and treat the disease; however, we believe that psychological intervention and counseling for the public are equally important.

A 59-year-old male presented to our clinic with intermittent foreign body sensation in both eyes for a few hours, without red eye or visual abnormalities. The patient complained of an intermittently “uncomfortable” feeling in his chest since taking a train through Wuhan city 7 days ago and indicated that it worsened when he read domestic or local news. He had no significant history of fever, cough, difficulty breathing, fatigue, or diarrhea. Ocular examination, radiological tests, and electrocardiograms showed no abnormalities, and the axillary temperature was 36.8°C. The patient suffered from symptoms with sleep disorders and anxiety intermittently; however, all symptoms were completely resolved after the end of the “14-day incubation period.” In addition, more patients sought care at our clinic with red eye, dry eye, or irritation in the eye, fearing “getting the virus.”

COVID-19 is a worldwide epidemic disease with rapid propagation, and the identified cases might only be the tip of the iceberg. A total of 835 confirmed cases with 25 fatalities were identified in Wuhan by January 24^4^; however, there are over 70 000 confirmed cases in China to date, including 1870 deaths, and several exported cases have been confirmed in other countries, such as Thailand, Japan, and the United States.^4^ Psychological panic is inevitable in this situation, and we hope that this letter will remind the global community that the emergence of this novel coronavirus requires treatments for physical ailments and a focus on science popularization of the disease to promote early psychological intervention and prevent the occurrence of psychogenic diseases.
